# Bonobos Share with Strangers

**DOI:** 10.1371/journal.pone.0051922

**Published:** 2013-01-02

**Authors:** Jingzhi Tan, Brian Hare

**Affiliations:** 1 Department of Evolutionary Anthropology, Duke University, Durham, North Carolina, United States of America; 2 Center for Cognitive Neuroscience, Duke University, Durham, North Carolina, United States of America; Max Planck Institute for Evolutionary Anthropology, Germany

## Abstract

Humans are thought to possess a unique proclivity to share with others – including strangers. This puzzling phenomenon has led many to suggest that sharing with strangers originates from human-unique language, social norms, warfare and/or cooperative breeding. However, bonobos, our closest living relative, are highly tolerant and, in the wild, are capable of having affiliative interactions with strangers. In four experiments, we therefore examined whether bonobos will voluntarily donate food to strangers. We show that bonobos will forego their own food for the benefit of interacting with a stranger. Their prosociality is in part driven by unselfish motivation, because bonobos will even help strangers acquire out-of-reach food when no desirable social interaction is possible. However, this prosociality has its limitations because bonobos will not donate food in their possession when a social interaction is not possible. These results indicate that other-regarding preferences toward strangers are not uniquely human. Moreover, language, social norms, warfare and cooperative breeding are unnecessary for the evolution of xenophilic sharing. Instead, we propose that prosociality toward strangers initially evolves due to selection for social tolerance, allowing the expansion of individual social networks. Human social norms and language may subsequently extend this ape-like social preference to the most costly contexts.

## Introduction

One of the most puzzling human behaviors from an evolutionary perspective is our species' propensity to share with non-relatives and even strangers [1], [2]. Across numerous cultures and early in development, humans engage in spontaneous helping and costly sharing with strangers [3], [4]. Some have suggested this human form of sharing is inconsistent with the predictions of kinship theory and reciprocal altruism (see [1], but see [5]) while others have proposed our species has evolved unique motivation and cognition for sharing [6]–[9].

Nonhuman primates are known to help and voluntarily share food with other groupmates (e.g. [10]–[16]). This prosociality, or voluntary behavior that benefits others [17]–[21], can be driven by selfish or other-regarding motivations [17], [22]. Therefore, while a primate can be prosocial even if pursuing selfish goals, they only demonstrate other-regarding forms of prosociality if their actions do not result in immediate selfish benefit (see SI for disambiguation of prosocial, other-regarding and altruistic behaviors). A number of experiments have now shown that a variety of primates will even help another individual obtain food when there is no immediate, tangible reward for their help (chimpanzees: [4], [23]–[27]; old world monkeys: [28]; new world monkeys: [29]–[31]). This type of prosociality suggests in some contexts primates also have other-regarding motivations (but see critique of this interpretation by [9]). However, there remains little evidence that nonhuman primates show any form of prosociality toward non-group members [7], [9], [13], [31], [32]. Primates typically compete against non-group members, resulting in agonistic intergroup relations [33]. This hostility goes to the extreme in chimpanzees that opportunistically kill neighbors [34], [35] and sometimes even immigrants [36]–[38]. Therefore, it is unlikely that most primates have tolerance levels that would allow for prosocial or other-regarding tendencies toward strangers. Moreover, designing such an experiment for most primate species would be extremely difficult given the high potential for stress, injury and aggression.

Bonobos are known for relatively high-levels of tolerance within and between groups when compared to chimpanzees [34], [39]–[43]. In the wild, bonobos have even been observed to have affiliative intergroup interactions. For example, females from neighboring communities have been seen traveling together for days, feeding in the same trees and even participating in socio-sexual behavior ([39], [40], also see [44]). In a preliminary experiment seven bonobos were given the opportunity to voluntarily share with another bonobo [12]. All three bonobos paired with a non-groupmate voluntarily shared their food while only one of the four bonobos paired with an in-group shared. No aggression of any form was ever observed. This suggests that with the relative tolerance of bonobos they can afford such prosociality with strangers. In turn, sharing with a stranger might aid them in extending their social network and in forming new “friendships” [5], [45]. However, it remains unclear whether the observed prosociality represents a preference to share with strangers over groupmates. In addition, it is unclear if the voluntary sharing observed only represents a selfish tactic to obtain a novel social interaction or whether bonobos will also share with strangers if there is no immediate, tangible reward. Therefore, we conducted four experiments with 15 wild-born bonobos that are orphans of the bushmeat trade living at Lola Ya Bonobo Sanctuary in Kinshasa, Democratic Republic of Congo [46]. We designed these experiments based on the relative costs and benefits of the prosocial behavior to the actor and this serial design allowed us to identify whether the prosocial motivation is selfish or other-regarding (Table 1). In experiment 1 and 2 we presented bonobos with a task in which they could choose whether to share food and physically interact with either a groupmate or stranger. In experiment 3 and 4 we presented bonobos with a second task in which they could either ignore or help another bonobo in obtaining out-of-reach food. In this second task helping allowed no immediate benefit to the actor (e.g. physical interactions) and the cost of helping was altered between experiment 3 and 4 (see Table 1).

**Table 1:** Summary of bonobo prosociality.

		Cost to the actor (food loss and/or energetic cost)	
		High	Low
Potential immediate benefit to the actor (a desirable physical interaction)	Yes	Stranger - Yes, Groupmate - No^1^	Stranger - Yes, Groupmate - Yes^2^
	No	Stranger - No, Groupmate - No^4^	Stranger - Yes, Groupmate - Yes^3^

1 . Prosociality driven by selfish motivation (i.e. self-regarding preferences): experiment 1–2 of current study; [12]; [47] also confirmed the groupmate results.

2 . Prosociality driven by selfish motivation (i.e. self-regarding preferences): The current series of experiments does not examine this type of prosociality since it does not require sharing. Given the results of experiment 1–2, this low-cost, high-benefit context does not allow us to examine the presence of any unselfish motivation. In a setup similar to experiment 1–2, [47] showed that when there was no food to share bonobos in a zoo opened a door for a groupmate, although they also opened the same door at similar rates in a non-social control (i.e. this suggests for groupmates, opening is probably not driven by social reward). We predict in the same contexts bonobos would open the door more frequently for a stranger than in a nonsocial control or for a groupmate since they do this in the current study when it results in the loss of food.

3 . Prosociality driven by unselfish motivation (i.e. other-regarding preferences): experiment 3 of current study.

4 . Prosociality driven by (stronger) unselfish motivation: experiment 4 of current study; [48] also confirmed the groupmate results.

## Experiment 1

**Figure 1:**
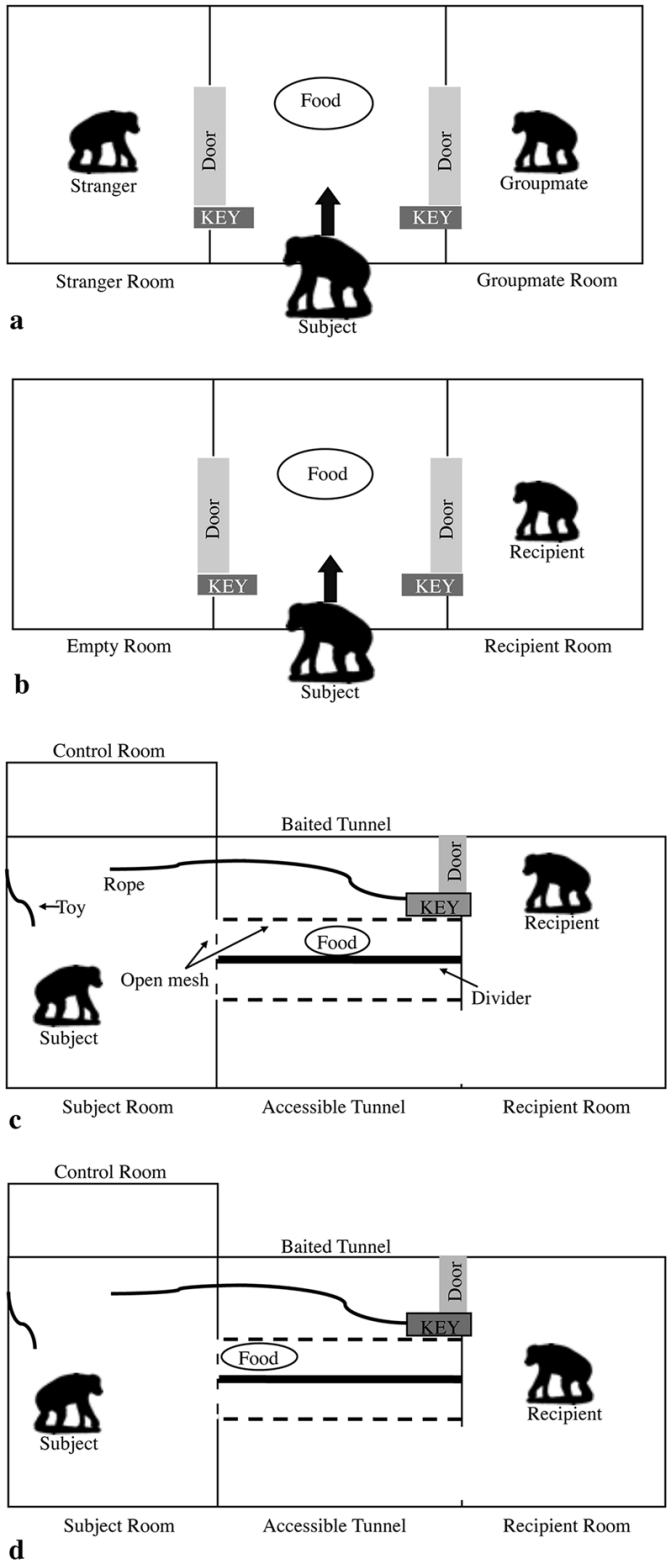
Experimental setups of experiment 1–4. In experiment 1 (**a**) and 2 (**b**), prosociality incurred a high cost (food loss) but potentially yielded immediate benefits (social interactions). In experiment 3 (**c**) and 4 (**d**), prosociality had no potential selfish benefits but always incurred a cost. Note that subjects always had complete control over the keys and therefore any prosocial behavior was voluntary.

The purpose of experiment 1 was to determine whether bonobos share and prefer to share food with strangers based on [12]. The subjects entered a room baited with a pile of highly desirable food. They could either eat all the food alone or they could co-feed with a conspecific by removing a one-way key to release either a groupmate or a stranger who were each locked in separate adjacent rooms (Figure 1a).

### Subjects

Fourteen bonobos (8F∶6M) from Lola ya Bonobo sanctuary participated in this experiment (see SI). All experiments were approved by the Ministry of Research in the Democratic Republic of the Congo (#MIN.RS/SG/004/2009), Lola ya Bonobo sanctuary and Duke IACUC. All subjects are orphans of bushmeat trade, but a comparison of their psychological health to mother-reared individuals revealed no substantial differences [49]. Each subject was tested with two conspecific recipients – one a stranger and the other a current groupmate. Seven female subjects played the role of the recipient (see Table S1 for pairings). We did not use male recipients simply because we did not have enough available at the time of the experiment. The composition of all trios allowed no role-reversal and maximized combinations of available recipients (see SI). Additionally, because pre-existing relationships among groupmates might be a confounding factor, we included as many individuals into the recipient pool as possible and randomly paired each subject with a groupmate recipient.

#### Strangers

were defined as unrelated individuals living in different social groups from one another. All subjects came from two different groups (see Table S1). Each group has a separate outdoor enclosure and set of indoor sleeping rooms. Strangers therefore did not have physical access to one another, because they were always physically separated by mesh and an electric fence. There was only possibility for vocal and visual communication, and this resembled the way wild bonobos from different populations interact [39]. Nine of fourteen of our stranger pairings were *complete strangers* who had never stayed in the same physical enclosure prior to the current experiment. We were able to examine individual records at the sanctuary to confirm which subjects were complete strangers. We tested the maximum number of complete strangers we could produce given sample size limitations and management constraints. Two pairings were not complete strangers because they met briefly during testing before they themselves were tested (i.e. they had served as recipients opposite one another for two previously tested subjects). For the last three pairings, they were former groupmates but had been transferred to different groups for at least one year (i.e. a period of time that in captive chimpanzees (*Pan troglodytes*) typically leads to a strong xenophobic response during reintegration attempts [50]).

### Setup

The experiment was conducted in three adjacent testing rooms (Figure 1a). These rooms (each 15 m^2^) were in the subjects' night building and were separated by open mesh. Manual sliding doors connected the middle room and the two side rooms where the recipients were placed for testing. The middle room also had a separate entrance (i.e. an overhead raceway) through which the subject could enter at the beginning of each test trial. A one-way key system was installed in each of the doors from the middle room into each of the side rooms. The keys consisted of wooden pegs that could be inserted on the subject's side of the door into a round metal hole in the track of the door. This blocked the path of the door unless the key was removed by the subject (see Figure S1a). Removing both keys simultaneously was impossible due to the distance between them. We thus created a setup in which bonobos in the middle room could determine whether to unlock a door(s) and which door to unlock first.

### Procedure

#### Food introduction

This was designed to demonstrate that subjects understood the one-way key system. One side room was baited with slices of apples or bananas and locked with the one-way key. Subjects had to successfully retrieve food out of the adjacent room in four out of five consecutive trials within 60 seconds.

#### No-food introduction

This was designed to demonstrate that subjects' door-opening was not simply intrinsically motivating but instead goal-directed. The setup was identical to the food introduction except food was placed in the middle room instead of in one of the side rooms. Subjects needed to inhibit removing the key for 60 s in four out of five consecutive trials in less than 21 trials.

#### Number pre-test

This was designed to demonstrate that subjects could make a choice between the contents of the two side rooms. Both side rooms were locked and one was baited with more food than the other. The locations of food were counterbalanced within and across subjects. Subjects had to first unlock the room with more food in four 1-minute trials of a five-trial session.

#### Test

For the test a potential recipient was moved into each of the two side rooms – one being a stranger to the subject (as well as the second recipient) and the other being a groupmate of the subject (see Figure 1a). The location of the different recipients were switched between trials and counterbalanced within and across subjects. Following [12], a mixture of food was placed in a small pile in the center of the food room (i.e. the middle room) beyond the reach of the recipients (see SI). A trial started when the subject entered the food room and ended when all the desirable food was claimed or seven minutes after the entry of the subject. Subjects were tested in a five-trial session with the same two recipients throughout, and they were tested early in the morning before their first meal to maximize their food motivation.

### Coding and analysis

Based on [15], [16], we define sharing as joint use of monopolizable food. Sharing is a type of prosocial behavior if it is voluntary, i.e. the possessor has the intention to allow the recipient access to food. However, this intention is not necessarily other-regarding or altruistic (i.e. instead they intentionally give another bonobo access to food without concern for the recipient's well-being).

As the measurement of sharing, door-opening was coded when a subject *first* removed the key to one of the doors but *only* if this occurred before all desirable food was *claimed*. Following [12], food being “claimed” was scored when a bonobo (both subjects and recipients) picked up each of the different pieces of food. This conservative criterion means only food that subjects did not pick up in the original food pile before releasing one of the recipients was scored as potentially sharable (i.e. food that subjects claimed but dropped might not represent their intention to share and would be excluded). Because a trial could take up to seven minutes, it was also possible for the second door to be opened releasing the second recipient before the end of the trial. A second door-opening was scored when either the subject or the first recipient removed the key to the second door - again only if this occurred before all the desirable food was claimed.

We coded food consumption if an individual placed food into its mouth. Because the bonobos could take a handful of food at once, we were unable to track the exact amount of food each recipient consumed. As a proxy, we compared “shared” feeding-time (i.e. from when a recipient was released until when all food was consumed) to total feeding-time (i.e. from when the subject started feeding to when all food was consumed). Socio-sexual behavior was scored when genital-genital contact occurred between two individuals once a recipient door was opened and before all the desirable food was claimed. Similarly, aggression was also scored if one bonobo fought with another bonobo resulting in screaming, hitting and biting. To assess the effect of recipients' solicitation, we categorized the recipient in each trial as either 1) *active* if they made any attempt to open the locked door or to reach the food, or 2) *passive* if no such behavior was observed. Inter-coder reliability was high (door-opening, food consumption, socio-sexual behavior, aggression: Cohen's K = 1; signaling behavior: K = 0.720; feeding time: *N* = 12, *r* = 0.993, Spearman's correlation). Nonparametric, two-tailed statistics were used in all analyses.

### Results

**Figure 2:**
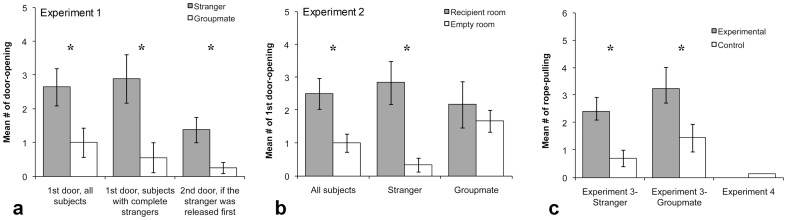
Results of experiment 1–4. ** *p*≤0.10, * *p*≤0.05, Wilcoxon test. In experiment 1 (**a**), we used two-tailed statistics. Based on the directional results of experiment 1 and those of [12], we had *a priori* predictions bonobos are spontaneously and preferentially prosocial toward strangers. Therefore, in experiment 2 (**b**) and 3–4 (**c**) we were justified to use one-tailed statistics.

See Figure 2a for results and Movie S1 for a sample video. The majority of the subjects (12 of 14) shared at least once and for a total of 51 trials (out of 70 or 72.9%). Subjects chose to release a complete stranger in preference to a groupmate before eating all the food (*N* = 9 (two ties), *Z* = 1.961, *p* = 0.05, Wilcoxon test), while having a strong tendency when all strangers are included (*N* = 14 (two ties), *Z* = 1.737, *p* = 0.081, Wilcoxon test). Nine subjects released the stranger first in more trials than the groupmate and only three subjects were in the opposite direction (see Table S1). Subjects also allowed the stranger but not the groupmate to co-feed for the majority of the total feeding time (stranger: *N* = 10, *T* = −2.090, *p* = 0.037; groupmate: *N* = 6, *T* = −0.105, *p* = 0.917, one-sample Wilcoxon signed rank test). Moreover, while unexpected, the second recipient was often released after the first even though there was remaining food that would need to be shared three ways. When the subject released the stranger first, the second recipient (the groupmate) was released by this first recipient (the stranger) more often than by the subject (*N* = 8 (one tie), *Z* = 1.983, *p* = 0.047, Wilcoxon test, Figure 2a).

Subjects consumed part of the food before releasing a recipient in 86.3% of trials where sharing occurred (44 of 51). The released recipients obtained desirable food in 78% of the trials (40 of 51). No form of aggression was ever observed. Socio-sexual behavior between the subjects and the first recipient released was observed in 20 trials (39.2%). This behavior only occurred between strangers but not groupmates (*N* = 51, *r* = 0.494, *p*<0.001, Phi coefficient). We found no co-variation between socio-sexual behavior and consumption of food by the recipient within trials where subjects unlocked a door (*N* = 51, *r* = 0.128, *p* = 0.360, Phi coefficient). Recipients' signaling behavior also did not correlate with subjects' tendency to share (*N* = 60, *r* = 0.074, *p* = 0.573, Spearman's correlation). Finally, subjects' prosociality did not change between the first and the last two trials (tendency to release a recipient: *N* = 14, *Z* = −0.378, *p* = 0.705; preference for releasing the stranger: *N* = 14, *Z* = −0.427, *p* = 0.669, Wilcoxon test).

### Discussion

Our results show that bonobos voluntarily share food with a recipient even when they could have monopolized it. They preferred to release the stranger and they allowed the stranger but not the groupmate to co-feed for the majority of total feeding time. Also, the surprising finding that the strange recipients voluntarily allowed a second recipient that was also strange to them into the same room (i.e. letting themselves be outnumbered by strangers) contrasts sharply with the xenophobic response of wild chimpanzees (i.e. wild chimpanzees rapidly retreat if they do not outnumber strangers by a factor of three; see [51]).

The subject's door opening was not a result of an inability to inhibit opening the door or inhibit interacting with the recipient. First, door-opening itself was not intrinsically motivating because in the no-food introduction subjects did not remove the key when there was no incentive to do so. Second, a preference for a specific recipient type is not predicted if door-opening alone motivated their choices. Third, bonobos are as capable of inhibiting door-opening as chimpanzees and 4–5-year-old children if it leads to food loss [52]. Fourth, Hare and Kwetuenda [12] previously demonstrated that some of these same bonobos tested again here do not open a door while eating food in the test room when other attractive items are in one of the adjacent rooms (i.e. additional food). Therefore, the subject's behavior was a voluntary choice to release the recipient over immediate feeding. As a result they intentionally forfeited some of the monopolizable food to the recipient (regardless of whether this sharing was selfishly or unselfishly motivated).

Subjects all passed the pretests and showed no temporal change in door-opening, which suggests that they clearly understood the consequence of opening the door. Subjects also did not open the door and then simply monopolize all the food. After being released by the subject, recipients consumed food in the majority of the trials (78%). Subjects were also highly food motivated since in 86.3% of trials they ate some food before sharing. In addition, we used an amount of desirable food that we knew subjects eat in its entirety based on a previous non-social control test (see [12]; we facilitated this by testing subjects before their morning meals). Subjects' door opening cannot be explained by tolerated theft or sharing-under-pressure, because the subjects had complete control over the food. No physical harassment was possible and no aggression was ever observed. The signaling behavior of the recipients also did not influence the subjects' sharing preference.

Reciprocal altruism is also not a plausible explanation for these results. First, there were no role reversal between subjects and recipients. This eliminates the possibility for tit-for-tat within the experiment. Second, reciprocal exchange before or after the testing period was impossible between non-groupmates. Third, contingent interchange of food-for-reproductive sex is not supported. Intercourse between a tumescent female and male was never observed. Non-reproductive socio-sexual behavior occurred at a low rate (39.2% of sharing trials). All of this occurred between female-female dyads or males and detumescent, pre-pubertal juveniles. Although socio-sexual behavior only occurred between stranger pairs, it did not correlate with food consumption by the recipient. Therefore, socio-sexual behavior was likely a by-product of sharing instead of the motivation behind the sharing behavior (also see [53]).

Experiment 1 replicated the findings of [10] that bonobos voluntarily chose to share monopolizable but highly desirable food with one another, including strangers. It further confirmed that bonobos have a xenophilic preference toward strangers over groupmates when sharing food. However, it was unclear whether this was caused by an inclination to share with strangers and/or a tendency to avoid groupmates. We adopted a between-subject design in experiment 2 to address this question.

## Experiment 2

In experiment 2, only one recipient was placed in one of the two adjacent rooms leaving the second adjacent room empty (see Figure 1b). For half of the subjects the potential recipient was a groupmate while for the other half she was a stranger. If the subjects were motivated to share, they should unlock the recipient room more often than the empty room.

### Methods

Because the current experiment examined the preference of door-opening instead of its occurrence, we tested all twelve bonobos (8F∶4M) that participated in experiment 1 that opened a door in at least one trial (see Table S1). Six subjects were paired with a groupmate and six with a stranger (five with a complete stranger). The location of the recipient was counterbalanced within subject.

The setup of experiment 2 was identical to experiment 1 with the exceptions that only one recipient was placed in one of the side rooms (leaving the other side room empty) and no pretests were conducted since this experiment was conducted days after the completion of experiment 1. In addition, having the empty room in this experiment served as an internal, non-social control for the intrinsic value of opening doors [12].

Strangers and behaviors were defined as in experiment 1. Nonparametric tests were applied throughout. Given the results of experiment 1 and of [12] showing prosocial sharing and a preference to share with strangers in bonobos, one-tailed statistics were used in comparing 1) rates of opening the recipient's door and the empty room and 2) rates of releasing the recipients between the two groups of subjects. All other analyses were two-tailed. Our primary measures followed those in experiment 1. Inter-coder agreement was high (feeding-time: *N* = 9, *r* = 0.987, Spearman's correlation; all other measures, Cohen's K = 1).

### Results

The majority of the subjects (11 of 12) unlocked the recipient at least once and for a total of 30 trials (out of 60 or 50%). Overall, the subjects unlocked the recipient door first more often than the empty room (*N* = 12 (two ties), *Z* = 1.955, *p* = 0.026, Wilcoxon test, one-tailed, Figure 2b and Movie S2). However, subjects only first opened the recipient's door more than the empty door when the recipient was a stranger (groupmate: *N* = 6 (one tie), *Z* = 0.552, *p* = 0.291; stranger: *N* = 6 (one tie), *Z* = 2.023, *p* = 0.022; complete stranger: *N* = 5 (one tie), *Z* = 1.890, *p* = 0.030, Wilcoxon test, all one-tailed, Figure 2b). When comparing the difference score between the rates of opening each door, subjects paired with a stranger again showed a stronger preference for unlocking the recipient door than those paired with a groupmate (all strangers: *N* = 12, *U* = 5.5, *p* = 0.021; pairs of complete strangers: *N* = 11, *U* = 5.5, *p* = 0.041, Mann-Whitney test, all one-tailed). Consistent with experiment 1, subjects again released strangers such that they could eat for the majority of the total feeding time, but here they also did the same for their groupmate (stranger: *N* = 6, *T* = −2.207, *p* = 0.014; groupmate: *N* = 5, *T* = −2.023, *p* = 0.022, one-sample Wilcoxon signed rank test). Subjects consumed some of the food before sharing in 76.7% (23 of 30) of trials. Recipients were able to eat food in the 80% of trials once released. Socio-sexual behavior was only observed in nine trials (of 30 sharing trials) in four stranger pairings and one groupmate pairing. It only occurred between female-female dyads and male-juvenile-female dyads. Again subjects' tendency to share neither correlated with recipient's request (*N* = 60, *r* = 0.052, *p* = 0.694, Spearman's correlation, two-tailed) nor changed between the first two and the last two trials (*N* = 12 (six ties), *Z* = −0.816, *p* = 0.414, Wilcoxon test, two-tailed).

### Discussion

The results of experiment 2 further support the idea that sharing was voluntary, prosocial and xenophilic. Subjects made a clear choice to share monopolizable food with strangers, while they were indifferent regarding groupmates (i.e. they did not avoid or approach groupmates). We thus confirmed that the results in experiment 1 were driven by an inclination to share with strangers.

Again subjects' behavior suggests door-opening was not simply caused by a lack of inhibitory control, because subjects opened doors according to the identity of the recipient not just the presence of a conspecific. Subjects were also food motivated since they ate in 76.7% of trials prior to sharing. Despotism cannot explain the results since the released recipients indeed ate food 80% of time. Interchange of food-for-sex is again not supported. Socio-sexual behavior occurred at a low frequency (30%) and had no reproductive function.

The results of the first two experiments show that bonobos are prosocial toward strangers, because the observed sharing was both voluntary and beneficial to others [17]. Subjects intentionally provided the recipient access to food by opening the door. They did this repeatedly across trials even though in other nonsocial contexts they quickly learn to avoid choices that lead to the loss of much smaller amounts of food [52], [54]. However, this willingness to relinquish food to others could be driven by two possible motivations (see Table 1). First, bonobos may only share food to facilitate a physical interaction with a stranger. Essentially, this type of food sharing is analogous to a form of tool-use where food sharing selfishly functions as a way to access a stranger. This predicts that the reward of initiating a novel interaction is so high that bonobos are willing to give up desirable food in exchange. However, if this alone motivates bonobos sharing they will not share when a physical interaction is impossible. Second, the observed sharing may in part be driven by other-regarding preference, an unselfish motivation based on concerns with other's welfare. This possibility is suggested by the fact that the food-motivated subjects could have easily monopolized all the food before releasing a recipient to interact. Instead, they chose to share. This motivational hypothesis predicts that bonobos will continue sharing with others even in contexts where a physical interaction is not possible. To test for the relative contribution of these motivational explanations, we designed a helping task in experiment 3 and 4 that allowed no physical interaction between participants. As a result, there was no immediate benefit for behaving prosocially, while the cost of helping was altered between experiment 3 and 4 (Table 1).

## Experiment 3

The purpose of experiment 3 was to determine whether bonobos are prosocial to strangers even if there is no immediate, tangible benefit. Subjects could pull a rope to release a recipient (a stranger or a groupmate) to acquire out-of-reach food. To raise the cost of the prosocial act, a novel toy was placed in the subjects' room so that helping also required forfeiting time playing. Importantly, the subject and the recipient were always physically separated, and the subject had no way to bring the recipient any closer (see Figure 1c).

### Subject

Ten bonobos (5F∶5M) participated in this experiment (see Table S2). Subjects were chosen based on their spontaneous level of comfort in the current experimental set-up (i.e. not all subjects were comfortable playing in the tunnels). All except one (Chibombo) had been tested in experiment 1 and 2 over a year before the start of this experiment. All except Sake were separately tested with both a stranger and a groupmate recipient. We were only able to pair Sake with a stranger due to time and space limitations. Of all 10 subject-stranger pairs, 7 were complete strangers. Recipients could be either female or male, but the stranger and the groupmate of any one subject were sex-matched. As in the previous two experimenters no reciprocity could occur between the subject and the recipient based on how recipients were assigned.

### Setup

The experiment was conducted in the subject room and the recipient room that were connected by two parallel tunnels (see Figure 1c). In addition, a control room (i.e. an overhead raceway) was adjacent to the subject room. In both tunnels the door to the recipient room could be locked with a one-way key installed inside the tunnel. The key was attached to a rope extending into the subject's room allowing subjects to potentially unlock the door. A divider was installed between the tunnels. A bonobo in one tunnel could reach through the tunnel mesh into the space between the divider and the tunnel, but they could not reach through the divider into the other tunnel area. This prevented recipients from obtaining food placed next to one tunnel from the opposite tunnel (see also Figure S1b).

### Procedure

#### Self-regard pre-test

This was designed to test whether subjects understood the physical set-up of the task. In this pre-test, subjects had to open one of the tunnels so they themselves could access the out-of-reach food (i.e. showing self-regard). The doors to both ends of one tunnel (the accessible tunnel) were open, which allowed the subject to travel between the two rooms (see Figure S2a). The other tunnel was baited with food and locked by the one-way key. Two slices of banana were placed in the space between this tunnel and the divider, and they were thus inaccessible from either the subject or the recipient room. The tunnel in which the food was placed was counterbalanced between trials. In order to enter the baited tunnel to retrieve the food, the subject had to pull the rope in the subject room and then travel through the accessible tunnel to open the door in the recipient room. Once the subject solved this problem on five consecutive trials within 60 seconds, they could proceed to the next pre-test.

#### No-food introduction

This session was designed to demonstrate that subjects did not simply find key removal intrinsically motivating (see Figure S2b). The configuration of the baited tunnel remained the same as the self-regard pre-test with the major exception that subjects had no possibility of retrieving the food (i.e. five banana pieces). As before, the door from the accessible tunnel into the recipient room was left open; whereas the door from this same tunnel and the subject room remained locked (such that removing the key blocking the door between the food tunnel and the room opposite to the subject room would not help subjects in obtaining the food, see SI). In addition, an attractive novel toy (a rope with a PVC tube attached) was placed in the subject room to provide an alternative activity to helping (based on [4], [23]). Finally, no other bonobos were present in any room adjacent to the subject room during this test. To proceed to the test on each of the two testing days (see below), subjects needed to inhibit pulling the rope for 60 s in five consecutive trials.

#### Test

Subjects were tested with the two different recipients on a separate day. The order of this testing was counterbalanced across subjects. For each recipient subjects were first tested in the no-food introduction and then received six experimental trials and six control trials in a block design. The order conditions were administered was counterbalanced across subjects. This means subjects received 12 test trials on each day or a total of 24 test trials. This design was used when the subject was tested with both the groupmate and the stranger. The order in which the stranger or groupmate recipient was paired with the subject was counterbalanced between subjects.

As seen in Figure 1c, the procedure of these trials were identical to the non-food introduction with the exception that in the experimental condition another bonobo was present in the recipient room and during the control condition the same recipient was in a room adjacent to the subject (the control room). As a result the subjects and the recipient were always physically separated. It is also important to note that pulling in the experimental condition could never bring the recipient in closer proximity, because it could always enter the accessible tunnel. Therefore, helping could not be motivated by the potential for a physical social interaction. In addition, a recipient was always present in a room adjacent to the subject room in both conditions. Therefore, unlocking the tunnel could not be explained by social facilitation (i.e. this followed the design of [4], [23]).

### Coding and analysis

Our main measure was rope-pulling. We scored a *rope-pull* when subjects pulled the rope attached to the key causing the key to be removed from the door within 60 s. We also coded a number of other behaviors to assess whether subjects' rope-pulls were somehow contingent on the behavior of the recipient. To assess the possibility of local enhancement caused by the recipient's positioning behavior, we coded how often a recipient was directly behind the locked door to the baited tunnel while the subject was looking on from behind their door to the same tunnel. Although the subject and the recipient were always separated by mesh, we scored social contact if there was any affiliative behavior (hugging, grooming, tickling and touching genitals) between the mesh. Signaling behavior was coded based on the same definition used in experiment 1 and 2. Inter-coder agreement was high (rope-pull: K = 0.906; local enhancement: K = 0.781; social contact: K = 0.841; signaling: K = 0.933). All statistics were nonparametric. Based on the prosociality observed in experiment 1 and 2, directional predictions were made and one-tailed statistics were used to compare 1) between the experimental and control conditions, 2) between subject's behavior with stranger and groupmate recipients. All other statistics were two-tailed.

### Results

The majority of the subjects (9 of 10) helped the recipient at least once (see Movie S3). Subjects pulled the rope in the experimental condition more often than in the control for both the stranger and the groupmate (stranger: pulling rate in the experimental condition = 40±8.7%, in the control condition = 11.7±5%, *N* = 10 (two ties), *Z* = −2.263, *p* = 0.012; groupmate: pulling rate in the experimental condition = 53.7±13.3%, in the control condition = 24±8.4%, *N* = 9 (one tie), *Z* = −2.257, *p* = 0.012, Wilcoxon test, all one-tailed, Figure 2c). They also helped the two categories of recipients equally often (*N* = 9 (four ties), *Z* = −0.137, *p* = 0.446, Wilcoxon test, one-tailed). The subjects' other-regarding preference did not vary with the sex of the recipient (stranger: *N* = 10, *U* = 5.5, *p* = 0.136; groupmate: *N* = 9, *U* = 8.5, *p* = 0.151, Mann-Whitney U test, two-tailed). However, the subjects' other-regarding preference was more xenophilic when the recipients were female than male (*N* = 9, *U* = 1.5, *p* = 0.029, Mann-Whitney U test, two-tailed, see Table S2). Male and female subjects did not differ in their tendency to help a recipient (stranger: *N* = 10, *U* = 10.5, *p* = 0.690; groupmate: *N* = 9, *U* = 6, *p* = 0.413, Mann-Whitney test, two-tailed) or their preference for helping a specific recipient (*N* = 9, *U* = 5.5, *p* = 0.247, Mann-Whitney test, two-tailed, Table S2).

The subjects' tendency to pull did not change between the first and the second half of a 12-trial session in one testing day (*N* = 10 (five ties), *Z* = −0.816, *p* = 0.414, Wilcoxon test, two-tailed), or when comparing their pulling rates between the first and the second recipient with which they were paired (i.e. between two testing days, *N* = 9 (four ties), *Z* = −0.412, *p* = 0.680, Wilcoxon test, two-tailed). The subjects' likelihood of pulling was not related to whether the recipient was directly behind the locked door or not (*N* = 8 (one tie), *Z* = −0.25, *p* = 0.799, Wilcoxon test, two-tailed). Helping did not increase the subjects' chances of having between-mesh social contact with the recipient (*N* = 9 (one tie), *Z* = −1.402, *p* = 0.161, Wilcoxon test, two-tailed). In addition, they were less likely to respond to an active than a passive recipient (chances of helping an active recipient: 41.86±12.25%; a passive recipient: 80.56±16.34%, *N* = 6 (one tie), *Z* = −2.023, *p* = 0.043, Wilcoxon test, two-tailed).

### Discussion

These findings show that even when there was no immediate social reward, bonobos are still motivated to help a stranger acquire out-of-reach food. Unlike experiment 1 and 2, not only strangers but also groupmates can become recipients of this prosocial act. Moreover, this prosociality could be directed to both male and female recipients, although subjects were more xenophilic toward females. These results do not support the hypotheses that other-regarding preference toward strangers is completely unique to humans [1], [7], [9], [13]. The sharing behavior of bonobos at least in part seems to be motivated by other-regarding preferences in addition to the desire to physically interact with strangers. Several low-level alternatives can be ruled out. Subjects all passed the self-regard pre-test, demonstrating clear understanding of the physical setup. They were always separated from the recipient and were not harassed into helping. Learning is also an implausible explanation. First, subjects' behavior did not change over time in the test. Second, all subjects passed the no-food introduction (i.e. no pulling for five consecutive trials). To make sure that they clearly understood that the food could not be obtained, we conducted the experimental and control sessions immediately after this no-food introduction. Therefore, it is unlikely subjects were removing the key in the experimental and control sessions because they were trying to acquire the food for themselves.

Local enhancement (i.e. the proximity of the recipient to the food or keys) cannot explain the observed helping since it had no effect on the subjects' likelihood of rope-pulling. It is also unlikely that the subjects' rope-pulling was motivated by a desire to bring the recipient into closer proximity, because 1) unlocking the baited tunnel did not bring the recipient into closer contact with the recipient since the other tunnel already allowed the recipient to potentially approach the subject (Figure 1c), and 2) experiment 1 and 2 predict more helping of strangers than the groupmates if increasing proximity was the subjects' sole motivation for helping. In addition, releasing the recipient did not increase rates of social contact. Reciprocity is again unlikely since role-reversals did not occur during the test and no repayment before or after the test was possible between strangers.

Experiment 3 shows that bonobos are motivated to help strangers even when the prosocial act has no immediate benefit (i.e. a physical interaction) but incurs a cost (see Table 1). In experiment 4, we test whether subjects will continue to help when there is no immediate benefit and an even greater cost to helping (loss of one's food).

## Experiment 4

In this final experiment the same paradigm from experiment 3 was used with the exception that food was placed within the subject's reach so that if the recipient was released both individuals had equal access to the food (Figure 1d). Helping would require subjects to forfeit food in their possession and did not create an opportunity for physical interaction since the subject and recipient still remained in separate rooms.

### Methods

Seven bonobos (4F∶3M) participated in this experiment. All were subjects from experiment 3 (see SI). Four were paired with a stranger and three with a groupmate (Table S2). The experimental design was identical to experiment 3 with the major exception that the baited food was moved within reach of the subject (see Figure 1d and SI). Subjects could easily reached through their door into the tunnel and eat the food or they could choose to release the recipient and eat the food together. All behavioral measures were the same as those used in experiment 3. Cohen's K of the recipient's behavior was 0.895.

### Results and Discussion

No subject ever released a recipient in an experimental trial (e.g. Movie S4). A single subject opened the door in one control trial. The refusal to release the recipient was not due to a loss of skill at opening the doors since subjects again passed a pre-test and again showed self-regard before the experiment began (see SI). This lack of helping also was not in response to a decrease in the recipient's requesting behavior, since it did not differ between experiment 3 and 4 (the recipient's chances of requesting in experiment 3: 89.29±6.98%; experiment 4: 69.05±8.47%, *N* = 7 (no tie), *Z* = −1.439, *p* = 0.15, Wilcoxon test, two-tailed). Instead the same subjects who helped in experiment 3 refused to share in experiment 4.

Although prosociality in experiment 1–2 and experiment 4 both incurred a high cost of food loss, sharing did not occur when subjects had no access to the recipient. This suggests that the xenophilic sharing observed in experiment 1–2 was in part motivated by a desire to initiate a physical interaction with the stranger (with potential for full body contact), and the payoff of this interaction was so high that they were even willing to forfeit highly desirable food to facilitate it (see Table 1). In experiment 4 there was so little opportunity for physical interaction that the benefit of the interaction no longer outweighed the cost in food (i.e. subjects could only potentially reach hands and feet through the bars to touch). As a result, subjects no longer shared with groupmates or strangers.

### General discussion

Our results demonstrate that prosociality and even other-regarding preferences toward strangers are not unique to humans. Our results also raise the possibility that bonobos have a unique prosocial preference for strangers over groupmates (i.e. while humans share with strangers they do not prefer them over groupmates [55], [56]). Our findings highlight two distinct motivations underlying prosociality toward strangers (see Table 1). First is a xenophilic motivation. In experiment 1 and 2 bonobos are willing to forego food in their possession to facilitate an interaction with a stranger – even preferring a stranger to a groupmate. However, this type of xenophilic sharing has limits. In experiment 4 bonobos will not give up valuable food in their possession unless a desirable social interaction is possible [48]. This supports the hypothesis that the relatively high tolerance observed in bonobos allows them to potentially extend their social networks through interactions with strangers [57], [58]. However, bonobo sharing is not completely selfishly motivated either. We also discovered a second, unselfish motivation toward strangers. In experiment 3 bonobos do exhibit other-regarding tendencies when no immediate payoff is available. Bonobos will exert effort to help strangers (and groupmates) obtain out-of-reach food as long as the cost of such helping is relatively low (i.e. does not require giving up food in their possession).

Controls demonstrate that the bonobos understood the physical properties of the two tasks (i.e. by demonstrating self-regard in a non-social pre-test) and were not opening doors due to local enhancement or a lack of inhibitory control. The observed sharing also cannot be explained by social factors including: harassment, since only subjects could allow recipients to approach the food; kinship, since no participant is related; repayment, since no reciprocal exchange before or after the experiment could occur between non-groupmates; and solicitation, since subjects' door opening behavior is not related to the requests of the recipients.

We predict future research with other captive bonobo populations will show a similar tendency for prosociality toward strangers since wild bonobos have the potential to affiliate with neighboring groups [39] and comparisons between the sanctuary bonobos and other captive bonobo populations have shown similar results in other cognitive domains [49]. Correspondingly, the xenophobia observed in captive chimpanzees mirrors the lethal aggression they can show toward neighboring groups in the wild (i.e. introducing chimpanzees to a pre-existing group often leads to serious injury and even fatalities; [50], [59]). It is also unlikely that bonobo's attraction to strangers is an expression of a more general preference for risk and novelty, since bonobos are more risk averse in foraging contexts [60] and more neophobic in non-social contexts [61] than chimpanzees. However, we also predict that future research will likely find variation in xenophilic sharing among bonobos depending on the age and sex combination of the actor and recipient. Throughout our experiments the majority of our subjects were juveniles and young adults (<15 years old; see Table S3 showing age of sexual maturity for sanctuary bonobos is between 7–8 years of age). In addition, the recipients in experiment 1 and 2 were always female. It is likely that older bonobos or even male-male pairings of bonobos will not show the same xenophilic preference observed in experiment 1 and 2. Given the variance observed in social behavior across different populations of wild chimpanzees [62] it is also possible that some chimpanzee pairings might show a xenophilic preference (i.e. male actors might prefer strange, adult female recipients). If an ethical way to test chimpanzees could be designed it would be interesting to know when and if they ever show a xenophilic preference for sharing with conspecifics (see [61] for evidence of xenophilia towards humans in chimpanzees). Another important future extension of the current work would be to test whether bonobos are more or less willing to share with groupmates based on their relationship quality during their natural group interactions. It may be that bonobos do readily volunteer to share with specific groupmates even though they do not prefer to share with all groupmates.

The current findings suggest that prosociality and even other-regarding behavior toward strangers is likely constrained across species by intergroup tolerance. Therefore, xenophilic prosociality is present in a species without language, social norms, intergroup violence or cooperative breeding because the benefits of initiating a new “friendship” and therefore expanding individual social network [45], [57], [58], [63] outweighed the costs of a prosocial interaction with a stranger (e.g. lethal aggression or feeding competition) [34], [64]. With little chance of serious conflict arising from intergroup interactions bonobos can more quickly develop positive relationships with non-group mates than groupmates with whom they have a long history of interactions (i.e. more social effort is needed to improve an existing relationship than to establish a completely new relationship). Future research will be necessary to establish if the relatively pacific bonobo is unusual among nonhumans in this regard or whether other species behave similarly toward strangers [65]. In addition, it is possible that bonobos may provide costly help to strangers in other contexts (although a method to non-verbally test nonhuman preferences toward an anonymous social partner remains elusive precisely because anonymity relies on linguistic capabilities).

Our findings suggest that the initial step toward the evolution of prosociality toward strangers may be selection against xenophobia [43], [65], [66], instead of selection facilitated by xenophobic aggression [67]. As a result, bonobos may be unique among apes in preferring to interact with strangers over groupmates even at the cost of sharing food. For humans, an increase in social tolerance likely resulted in bi-sexual dispersal and an expanded social network of unrelated individuals [68], which further enabled cumulative culture and cooperation [6], [69]. Based on current evidence, it is likely that humans are unique for the ability to extend our ape-like prosociality even to the most costly of contexts. These extreme other-regarding preferences possibly rely on language and social norms making it unlikely that such preferences preceded the evolution of these socio-cognitive abilities [8].

## Supporting Information

Text S1 **Supplementary methods, results, discussion and references.**
(DOC)

Figure S1 **Photos of the setups of the four experiments: a) The one-way key system in experiment 1 and 2, viewed from the subject room.** **b**) The general setup of experiment 3 and 4. This particular photo shows the subject, the baited tunnel, the divider, the one-way key in the locked position and the food as it was placed in experiment 4.
(JPG)

Figure S2 **Setups of self-regard pre-test and no-food introduction of experiment 3–4:** **a) The self-regard pre-test of experiment 3 and 4.** b) The no-food introduction of experiment 3 and 4, during which no other bonobos were present in adjacent rooms. In both phases, the subjects had to meet the corresponding criteria in five consecutive trials to proceed to the next test phase.
(JPG)

Table S1 **Subject information of experiment 1 and 2.**
(PDF)

Table S2 **Subject information of experiment 3 and 4.**
(PDF)

Table S3 **Reproductive history of female bonobos from the sanctuary.**
(PDF)

Movie S1 **Subjects voluntarily chose to and preferred to release the stranger in experiment 1.**
(AVI)

Movie S2 **Subjects voluntarily released the stranger but not the groupmate in experiment 2.**
(AVI)

Movie S3 **Subjects helped the recipient to acquire out-of-reach food in experiment 3.**
(AVI)

Movie S4 **Subjects did not release the recipient in experiment 4.**
(AVI)

## References

[1] Fehr E, Fischbacher U (2003) The nature of human altruism. Nature 425: 785–791. 14574401 10.1038/nature02043

[2] Seabright P (2004) The Company of Strangers. (Princeton University Press)

[3] Henrich J, Boyd R, Bowles S, Camerer C, Fehr E, et al (2005) “Economic man” in cross-cultural perspective: behavioral experiments in 15 small-scale societies. Behavioral and Brain Sciences 28: 795–815. 16372952 10.1017/S0140525X05000142

[4] Warneken F, Hare B, Melis A, Hanus D, Tomasello M (2007) Spontaneous altruism by chimpanzees and young children. PLoS Biology 5: e184. 17594177 10.1371/journal.pbio.0050184 PMC1896184

[5] Delton AW, Krasnow MM, Cosmides L, Tooby J (2011) Evolution of direct reciprocity under uncertainty can explain human generosity in one-shot encounters. Proceedings of the National Academy of Sciences 108: 13335–13340. 10.1073/pnas.1102131108 PMC3156224 21788489

[6] Tomasello M (2009) Why we cooperate. (MIT press).

[7] Burkart J, Hrdy SB, van Schaik C (2009) Cooperative breeding and human cognitive evolution. Evolutionary Anthropology 18: 175–186.

[8] Hill K, Barton M, Hurtado A (2009) The emergence of human uniqueness: characters underlying behavioral modernity. Evolutionary Anthropology 18: 187–200.

[9] Silk J, House B (2011) Evolutionary foundations of human prosocial sentiments. Proceedings of the National Academy of Sciences 108: 10910–10917. 10.1073/pnas.1100305108 PMC3131813 21690372

[10] Stevens JR (2004) The selfish nature of generosity: harassment and food sharing in primates. Proceedings of the Royal Society B: Biological Sciences 271: 451–456. 15129953 10.1098/rspb.2003.2625 PMC1691616

[11] de Waal FBM (1997) Food transfers through mesh in brown capuchins. Journal of Comparative Psychology 111: 370–378. 9419882 10.1037/0735-7036.111.4.370

[12] Hare B, Kwetuenda S (2010) Bonobos voluntarily share their own food with others. Current Biology 20: R230–231. 20219170 10.1016/j.cub.2009.12.038

[13] Cheney DL (2011) Extent and limits of cooperation in animals. Proceedings of the National Academy of Sciences 108: 10902–9. 10.1073/pnas.1100291108 PMC3131815 21690405

[14] Feistner A, McGrew W (1989) Food-sharing in primates: a critical review. In: Seth P, Seth S, editors. Perspectives in primate biology, Vol. 3. New Delhi: Today Tomorrow's. pp. 21–36.

[15] Stevens JR, Gilby IC (2004) A conceptual framework for nonkin food sharing: timing and currency of benefits. Animal Behaviour 67: 603–614.

[16] Jaeggi AV, Schaik CP (2011) The evolution of food sharing in primates. Behavioral Ecology and Sociobiology 65: 2125–2140.

[17] Eisenberg N, Fabes R, Spinrad T (2006) Prosocial development. In: Eisenberg N, eds, Handbook of Child Psychology. John Wiley & Sons. pp 646–718.

[18] Cronin K (2012) Prosocial behaviour in animals: the influence of social relationships, communication and rewards. Animal Behaviour 84: 1085–1093.

[19] Jaeggi AV, Burkart JM, Van Schaik CP, Schaik CPV (2010) On the psychology of cooperation in humans and other primates: combining the natural history and experimental evidence of prosociality. Philosophical transactions of the Royal Society of London Series B, Biological sciences 365: 2723–2735. 20679115 10.1098/rstb.2010.0118 PMC2936168

[20] House BR, Henrich J, Brosnan SF, Silk JB (2012) The ontogeny of human prosociality: behavioral experiments with children aged 3 to 8. Evolution and Human Behavior 33: 291–308.

[21] Vaish A, Warneken F (2011) Social-Cognitive Contributors to Young Children's Empathic and Prosocial Behavior. In: Decety J, eds. Empathy: From Bench to Bedside. Cambridge: MIT Press. pp 131–146.

[22] Fehr E, Bernhard H (2008) Rockenbach B (2008) Egalitarianism in young children. Nature 454: 1079–1083. 18756249 10.1038/nature07155

[23] Melis AP, Warneken F, Jensen K, Schneider A-C, Call J, et al (2011) Chimpanzee help conspecifics obtain food and non-food items. Proceedings of the Royal Society B: Biological Sciences 278: 1405–13. 20980301 10.1098/rspb.2010.1735 PMC3061135

[24] Greenberg JR, Hamann K, Warneken F, Tomasello M (2010) Chimpanzee helping in collaborative and noncollaborative contexts. Animal Behaviour 80: 873–880.

[25] Horner V, Carter JD, Suchak M, de Waal FBM (2011) Spontaneous prosocial choice by chimpanzees. Proceedings of the National Academy of Sciences 108: 13847–13851. 10.1073/pnas.1111088108 PMC3158226 21825175

[26] Yamamoto S, Humle T, Tanaka M (2009) Chimpanzees help each other upon request. PLoS One 4: e7416. 19826478 10.1371/journal.pone.0007416 PMC2757899

[27] Yamamoto S, Humle T, Tanaka M (2012) Chimpanzees' flexible targeted helping based on an understanding of conspecifics' goals. Proceedings of the National Academy of Sciences 1108517109v1–201108517. 10.1073/pnas.1108517109 PMC3295314 22315399

[28] Massen JJM, van den Berg LM, Spruijt BM, Sterck EHM (2010) Generous leaders and selfish underdogs: pro-sociality in despotic macaques. PLoS ONE 5: e9734. 20305812 10.1371/journal.pone.0009734 PMC2840023

[29] Burkart J, Fehr E, Efferson C, van Schaik C (2007) Other-regarding preferences in a non-human primate: common marmosets provision food altruistically. Proceedings of the National Academy of Sciences 104: 19762–6. 10.1073/pnas.0710310104 PMC2148372 18077409

[30] Lakshminarayanan V, Santos L (2008) Capuchin monkeys are sensitive to others' welfare. Current Biology 18: R999–R1000. 19000809 10.1016/j.cub.2008.08.057

[31] de Waal FBM, Leimgruber K, Greenberg AR (2008) Giving is self-rewarding for monkeys. Proceedings of the National Academy of Sciences 105: 13685–9. 10.1073/pnas.0807060105 PMC2533250 18757730

[32] Melis AP, Semmann D (2010) How is human cooperation different? Philosophical Transactions of the Royal Society B: Biological Sciences 365: 2663–2674. 10.1098/rstb.2010.0157 PMC2936178 20679110

[33] Crofoot M, Wrangham R (2010) Intergroup aggression in primates and humans: the case for a unified theory. In: Kappeler P, Silk J, eds Mind the Gap. Springer. pp 171–195.

[34] Wrangham R (1999) Evolution of coalitionary killing. Yearbook of Physical Anthropology 42: 1–30. 10.1002/(sici)1096-8644(1999)110:29+<1::aid-ajpa2>3.3.co;2-5 10601982

[35] Muller M, Mitani J (2005) Conflict and cooperation in wild chimpanzees. Advances in the Study of Behavior 35: 275–331.

[36] Kahlenberg S, Thompson M, Muller M, Wrangham R (2008) Immigration costs for female chimpanzees and male protection as an immigrant counterstrategy to intrasexual aggression. Animal Behaviour 76: 1497–1509.

[37] Pusey A, Murray C, Wallauer W, Wilson M, Wroblewski E, et al (2008) Severe aggression among female Pan troglodytes schweinfurthii at Gombe National Park, Tanzania. International J of Primatology 29: 949–973.

[38] Townsend S, Slocombe K, Emery Thompson M, Zuberbühler K (2007) Female-led infanticide in wild chimpanzees. Current Biology 17: R355–356. 17502085 10.1016/j.cub.2007.03.020

[39] Furuichi T (2011) Female contributions to the peaceful nature of bonobo society. Evolutionary Anthropology 20: 131–142. 22038769 10.1002/evan.20308

[40] Idani G (1991) Social relationships between immigrant and resident bonobo (Pan paniscus) females at Wamba. Folia Primatologica 57: 83–95. 10.1159/000156568 1786910

[41] Hohmann G (2001) Association and social interactions between strangers and residents in bonobos (Pan paniscus). Primates 42: 91–99.

[42] Wobber V, Wrangham R, Hare B (2010) Bonobos exhibit delayed development of social behavior and cognition relative to chimpanzees. Current Biology 20: 226–230. 20116251 10.1016/j.cub.2009.11.070

[43] Hare B, Wobber V, Wrangham R (2012) The self-domestication hypothesis: bonobo psychology evolved due to selection against aggression. Animal Behaviour 83: 573–585.

[44] Gold K (2001) Group formation in captive bonobos: sex as a bonding strategy. The Apes: Challenges for 21st Century. Brookfield Zoo, Brookfield. pp. 90–93.

[45] Noë R, Hammerstein P (1994) Biological markets: supply and demand determine the effect of partner choice in cooperation, mutualism and mating. Behavioral Ecology and Sociobiology 35: 1–11.

[46] André C, Kamate C, Mbonzo P, Morel D, Hare B (2008) The conservation value of Lola ya Bonobo Sanctuary. In: Furuichi T, Thompson J, editors. The Bonobos. Springer, Berlin Heidelberg. pp. 303–322.

[47] Bullinger AE, Melis AP, Tomasello M (in press) Bonobos, *Pan paniscus*, chimpanzees, *Pan troglodytes*, and marmosets, *Callithrix jacchus*, prefer to feed alone. Animal Behaviour http://dx.doi.org/10.1016/j.anbehav.2012.10.006.

[48] Jaeggi A, Stevens J, van Schaik C (2010) Tolerant food sharing and reciprocity is precluded by despotism among bonobos but not chimpanzees. American Journal of Physical Anthropology 143: 41–51. 20310060 10.1002/ajpa.21288

[49] Wobber V, Hare B (2011) Psychological health of orphan bonobos and chimpanzees in African sanctuaries. PLoS One 6: e17147. 21666743 10.1371/journal.pone.0017147 PMC3110182

[50] Seres M, Aureli F, de Waal FBM (2001) Successful formation of a large chimpanzee group out of two pre-existing subgroups. Zoo Biology 20: 501–515.

[51] Wilson M, Hauser M, Wrangham R (2001) Does participation in intergroup conflict depend on numerical assessment, range location, or rank for wild chimpanzees? Animal Behaviour 61: 1203–1216.

[52] Vlamings PHJM, Hare B, Call J (2010) Reaching around barriers: the performance of the great apes and 3–5-year-old children. Animal Cognition 13: 273–285. 19653018 10.1007/s10071-009-0265-5 PMC2822225

[53] Woods V, Hare B (2011) Bonobo but not chimpanzee infants use socio-sexual contact with peers. Primates 52: 111–116. 21127940 10.1007/s10329-010-0229-z

[54] Rosati A, Hare B (2012) Decision making across social contexts: competition increases preferences for risk in chimpanzees and bonobos. Animal Behaviour 84: 869–879.

[55] Fehr E, Bernhard H, Rockenbach B (2008) Egalitarianism in young children. Nature 454: 1079–1083. 18756249 10.1038/nature07155

[56] Levine M, Prosser A, Evans D, Reicher S (2005) Identity and emergency intervention: how social group membership and inclusiveness of group boundaries shape helping behavior. Personality & Social Psychology Bulletin 31: 443–453. 15743980 10.1177/0146167204271651

[57] Engh ACBJ, Bergman TJ, Whitten PL, Hoffmeier RR, Seyfarth RM, et al (2006) Behavioural and hormonal responses to predation in female chacma baboons (*Papio hamadryas ursinus*). Proceedings of the Royal Society B: Biological Sciences 273: 707–712. 16608690 10.1098/rspb.2005.3378 PMC1560071

[58] Taylor S, Klein L, Lewis B, Gruenewald T, Gurung R, et al (2000) Biobehavioral responses to stress in females: Tend-and-befriend, not fight-or-flight. Psychological Review 107: 411–429. 10941275 10.1037/0033-295x.107.3.411

[59] Brent L (2001) The Care and Management of Captive Chimpanzees. American Society of Primatologists, San Antonio, TX.

[60] Heilbronner S, Rosati A, Stevens J, Hare B, Hauser M (2008) A fruit in the hand or two in the bush? Divergent risk preferences in chimpanzees and bonobos. Biology Letters 4: 246. 18364305 10.1098/rsbl.2008.0081 PMC2610056

[61] Herrmann E, Hare B, Cissewski J, Tomasello M (2011) A comparison of temperament in nonhuman apes and human infants. Developmental Science 14: 1393–1405. 22010898 10.1111/j.1467-7687.2011.01082.x

[62] Stumpf R (2011) Chimpanzees and bonobos: diversity within and between species. In: Campbell CJ, Fuentes A, MacKinnon, Katherine C, Bearder SK, Stumpf RM, editors. Primates in Perspective. Oxford, New York: Oxford University Press. pp. 340–356.

[63] Silk JB (2007) Social components of fitness in primate groups. Science 317: 1347–1351. 17823344 10.1126/science.1140734

[64] Kappeler P, Schaik CV (2002) Evolution of primate social systems. International Journal of Primatology 23: 707–740.

[65] Ganem G, Bennett N (2004) Tolerance to unfamiliar conspecifics varies with social organization in female African mole-rats. Physiology & Behavior 82: 555–562. 15276822 10.1016/j.physbeh.2004.05.002

[66] Hare B (2007) From nonhuman to human mind: what changed and why? Current Directions in Psychological Science 16: 60–4.

[67] Choi J, Bowles S (2007) The coevolution of parochial altruism and war. Science 318: 636–40. 17962562 10.1126/science.1144237

[68] Hill K (2011) Co-residence patterns in hunter-gatherer societies show unique human social structure. Science 331: 1286–9. 21393537 10.1126/science.1199071

[69] Foley R, Gamble C (2009) The ecology of social transitions in human evolution. Philosophical Transactions of the Royal Society B: Biological Sciences 364: 3267–3279. 10.1098/rstb.2009.0136 PMC2781881 19805433

