# Supplementary material for: Bonobos Share with Strangers
Source: PLoS One. 2013 Jan 2;8(1):e51922. doi: 10.1371/journal.pone.0051922 (PMC3534679; doi:10.1371/journal.pone.0051922)
Supplement: Text S1 — Supplementary methods, results, discussion and references. (DOC) [file pone.0051922.s001.doc]

Supporting Information

Supplementary Discussion

Prosociality, other-regarding behavior, and altruism have been defined in a variety of ways [70]. We consider a behavior as prosocial if it benefits another individual, even if the actor is motivated selfishly [17-22]. Following Silk and colleagues [71], we consider helping behavior as other-regarding when no apparent or immediate benefit is received by the actor in return. Therefore, other-regarding behavior is one type of prosociality. Following West and colleagues [70], we consider helping to be altruistic if it incurs a cost to oneself. Therefore, altruism is a class of other-regarding behaviors that is also pro-social in nature.

Table 1 summarizes the results of experiment 1-4 and pertinent studies in a payoff matrix that illustrates the costs and (potential) immediate benefits of prosociality. Note that prosociality toward strangers in bonobos includes a xenophilic motivation (selfish), as shown by experiment 1, 2 and 4, as well as an other-regarding (or even altruistic) motivation, which can be seen in experiment 3. The current study is not aimed at examining the situation characterized of low cost and potential benefit (the top left cell in Table 1), because it is apparently the least challenging situation in the matrix. One example will be a “no-food” version of our experiment 1-2 [47]. Given bonobos are even willing to release the stranger at the cost of food loss, we predict that they will release the stranger (and perhaps more often) when no food is involved.

Due to the clear xenophilia observed in our sample (bonobos who never shared a physical enclosure but had limited visual and vocal communication), we predict future research with strangers of absolute unfamiliarity or even anonymity will at least demonstrate a similar xenophilic preference if not stronger.

It is also interesting to consider our results in light of the well-documented xenophobia in chimpanzees, the other species of *Pan*. Chimpanzees tend to be extremely hostile toward strangers: they show strong aversion to immigrants [36-38], cooperate in patrolling territorial borders and opportunistically kill neighbors [34-35]. Killing adult males and infants from neighboring groups allows chimpanzees to take over the territory and females of their neighbors [34,72]. Lethal intergroup aggression evolved as a competitive advantage over other groups, and is the largest contributor to adult mortality in chimpanzees [73]. Lone chimpanzees are, therefore, averse to the risk associated with encountering strangers [35,51,74,75]. In captivity, integrating new individuals is extremely challenging for management [50,59]. As a result, although it is well evident that chimpanzees are prosocial toward groupmates at a similar level of bonobos [4, 23-27], it is highly unlikely that they will direct any prosocial behavior to strangers, let alone having a xenophilic preference like bonobos. This contrast suggests that bonobos might provide a better model for us to understand the extreme prosociality toward strangers in humans.

General Methodology

A total of 15 semi-free ranging bonobos (8F:7M) from Lola Ya Bonobo Sanctuary in Kinshasa, Democratic Republic of Congo, participated in the four experiments. See Table S1-2 for age, sex and experimental history of each subject participating in each experiment. All subjects are orphans of bushmeat trade and live in one of three social groups with daily access to a large and rich forested enclosure (5-20 hectares). At night each group sleeps separately in night enclosures. They are fed four times a day with fresh fruits, vegetables and nuts.

Before leaving the night enclosures for the day, subjects were separated for the purpose of participating in the tests and were offered supplemental food. The subjects were never food-deprived and water was always available at all times during testing. All tests were voluntary. Subjects could quit at any time by sitting next to an exit to the building – to which they always had access. Any sign of distress during testing would be reason to release a participant back into the group. Subjects were chosen for the test based on their previous interest in participating in other behavioral testing and their spontaneous level of comfort in the current experimental set-up. We have no reason to suspect that the subjects we tested in the current experiment are not representative of the sharing behavior of other bonobos who did not participate in the current series of studies (i.e. subjects who are not included were not tested due to factors totally unrelated to their ability to share). For a more detailed description of the testing site, the rearing history, and psychological health of these sanctuary apes see [49].

In all experiments subjects were tested in familiar rooms of their night holding facilities (15 m²). Rooms were separated by mesh and manual sliding doors. To maximize subject’s food motivation, in all experiments they were tested early in the morning before their first meal. Twelve months before experiment 1 and 2, five subjects had participated in a study with similar door-opening design [12]. Largely based on the method of Hare and Kwetuenda, experiment 1 and 2 were then conducted in Spring 2010. Piloting for the method used in experiment 3 and 4 was then conducted in summer of 2010. Eight subjects participated in two brief pilot methods that we did not complete due to procedural limitations in our initial designs. Finally, in Summer 2011 experiment 3 and 4 were conducted.

Experiment 1

*Supplementary methods*

Three rules were used to decide the composition of each trio: (1) to exclude the possibility of reciprocity a bonobo never served as a recipient in a trio in which the subject had previously been that individual’s recipient; 2) to maximize combinations of trios different recipient pairs were created yielding 10 different recipient combinations in the 14 trios; 3) to avoid pairing individuals in which one individual might be in danger in the very unlikely event that an unexpected fight occurred we tested subjects with similarly sized recipients (i.e. we avoided pairing the smallest infants with much larger adults).

In the test, the food pile was comprised of approximately ten slices of apples (equal to a quarter apple), three slices of bananas (equal to half a banana), ten peanuts, three slices of papayas (equal to about one tenth of a papaya) and three slices of cucumber. Though all types of food were in a pile, they were arranged to aid their visibility to the experimenter. *Desirable* food referred to apple, banana, papaya and peanut [based on 12; Rosati & Hare, unpublished data].

All trials were videotaped by at least one camera. The food introduction, the no-food introduction and the number pre-test were all coded live because scoring the subjects’ removal of a key was unambiguous. The test trials were videotaped using two JVC cameras. The cameras were positioned such that each focused on one of the doors from the middle room into one of the side rooms.

*Supplementary results*

In food introduction, all subjects quickly passed the criteria. The mean number of trials needed for subjects to pass this criterion was 9.07 ± 1.48 (4 - 21 trials). In no-food introduction, all subjects tested quickly met this criterion (mean: 7.69 ± 1.27, 4-20 trials) with one exception (Kinshasa received a full 21 trials but then in the test went on to be one of the most inhibited in opening doors for her recipient). In number pre-test, 12 of 14 subjects passed spontaneously in the first session. Only two subjects (Masisi and Sake) required a second session to pass this criterion.

Irrespective of the recipient’s identity, in 12 of 14 trios (85.7%) the subjects unlocked the door before all the desirable food was claimed for at least one trial, yielding a sum of 51 (72.9%) trials of 70 total with food-sharing. A stranger was released first by 10 of 14 subjects (71.4%) at least once for a total of 37 trials (52.9% of all trials or 72.5% of the trials with food-sharing). The second recipient was released in 32 (62.7%) of the 51 trials where sharing occurred.

Subjects’ age correlated with neither their overall tendency to release a recipient (*N* = 14, *r* = -0.436, *p* = 0.119, Spearman’s correlation) nor their preference for a specific recipient (*N* = 12, *r* = 0.472, *p* = 0.121, Spearman’s correlation). Subjects’ sex did not affect their overall sharing tendency (*N* = 14, *U* = 17.5, *p* = 0.414, Mann-Whitney). But among those subjects who shared at least once, the preference for strangers was stronger in males relative to females (*N* = 12, *U* = 4, *p* = 0.048, Mann-Whitney).

In 11 cases where the recipient did not eat food after being released, nine were caused by the reticence of a particular recipient (Waka) to eat and were not due to the subject. This is evidenced by the fact that Waka opened the groupmate’s door first more than the stranger’s door – as one of the youngest individuals tested she was likely nervous about interacting with strangers.

Experiment 2

*Supplementary results*

Subjects’ age did not correlate with sharing (*N* = 12, *r* = -0.132, *p* = 0.683, Spearman correlation). Unlike experiment 1, the sex of the subjects did not affect the tendency to share with any recipient (*N* = 12, *U* = 11, *p* = 0.461), with a stranger (*N* = 6, *U* = 2.5, *p* = 0.533), or with a groupmate (*N* = 6, *U* = 3, *p* = 0.800).

Experiment 3

*Supplementary methods*

Note that because this experiment was conducted 15 months after experiment 2, the group membership of some subjects changed (Table S2). In the test, one experimenter (E1) baited the food tunnel and positioned the key while a second experimenter (E2) distracted the subject with juice. A trial started as E2 placed 5-15 tiny pieces of apple in the far corner of the subjects’ room (away from the tunnels) and left the testing area with E1 (this was done so that the experimenter’s departure did not distract the subjects).

*Supplementary results*

In self-regard pre-test, subjects quickly passed this test showing a solid understanding of the physical set-up. Mean number of trials needed to pass this phase was 5.90 ± 0.71 (5 - 12 trials). Therefore, subjects’ door-opening in the test phase cannot be explained as being due to a lack of understanding the contingencies of the physical set-up. In no-food introduction, subjects only needed a mean of 11.50 ± 2.04 (5 - 26 trials) to pass. Age of subjects again did not co-vary with helping (stranger: *N* = 10, *r* = -0.207, *p* = 0.567; groupmate: *N* = 9, *r* = -0.507, *p* = 0.164).

Experiment 4

*Supplementary methods*

Three subjects of experiment 3 were not included because one (Masisi) never pulled the rope once in experiment 3, one (Kasongo) became unwilling to be separated, and one (Chibombo) did not pass the self-regard pre-test in experiment 4 before the end of the field trip.

Experiment 4 was identical to experiment 3 with the exceptions that: (1) the food was placed within the subjects’ reach in the no-food introduction, experimental and control phases; (2) in the experimental and control conditions, the amount of food increased to 30 pieces, including 20 papaya (about 1/12 of one papaya), 5 banana (about 1/4 banana) and 5 apple (about 1/6 apple) pieces; (3) experimental and control trials were 5-minute long, and rope-pulling was coded if a subject pulled the key within 5 minutes of the trial starting or before all the food was claimed. Subjects needed a mean of 5.29 ± 0.18 trials to pass the self-regard pre-test and a mean of 6.57 ± 0.90 trials to pass the no-food introduction.

*Supplementary results*

The subjects never pulled the rope in the experimental condition, while only one subject pulled the rope in a single control trial (Figure 2d, Table S2). The results of experiment 4 were also not a product of learning (or extinction of the pulling behavior). All subjects passed the self-regard pre-test again before this experiment in less than seven trials. This demonstrates subjects’ still understood the properties of the one-way key system. The inhibition phase was also unlikely to completely suppress subjects’ pulling behavior, because in experiment 3, they were willing to help after they received the same inhibition phase prior to the experimental and control sessions (note: they also had access to a toy and a much smaller amount of food during each trial of experiment 3).

Supplementary Reference

1. West, S., Griffin, A. & Gardner, A. 2007 Social semantics: altruism, cooperation, mutualism, strong reciprocity and group selection*.* Journal of Evolutionary Biology 20: 415-432
2. Silk, J., Brosnan, S., Vonk, J., Henrich, J., Povinelli, D., Richardson, A., Lambeth, S., Mascaro, J. & Schapiro, S. 2005 Chimpanzees are indifferent to the welfare of unrelated group members. Nature 437: 1357-1359
3. Mitani, J., Watts, D. & Amsler, S. 2010. Lethal intergroup aggression leads to territorial expansion in wild chimpanzees. Current Biology 20: R507-508.
4. Williams, J., Lonsdorf, E., Wilson, M., Schumacher-Stankey, J., Goodall, J. & Pusey, A. 2008. Causes of death in the Kasekela chimpanzees of Gombe National Park, Tanzania. American Journal of Primatology 70: 766-777.
5. Wilson, M., Hauser, M. & Wrangham, R. 2007. Chimpanzees (Pan troglodytes) modify grouping and vocal behaviour in response to location-specific risk. Behaviour 144: 1621-1653.
6. Emery Thompson, M., Kahlenberg, S., Gilby, I. & Wrangham, R. 2007. Core area quality is associated with variance in reproductive success among female chimpanzees at Kibale National Park. Animal Behaviour 73: 501-512.
