# Supplementary figures and images for: Bonobos Share with Strangers
Source: PLoS One. 2013 Jan 2;8(1):e51922. doi: 10.1371/journal.pone.0051922 (PMC3534679; doi:10.1371/journal.pone.0051922)

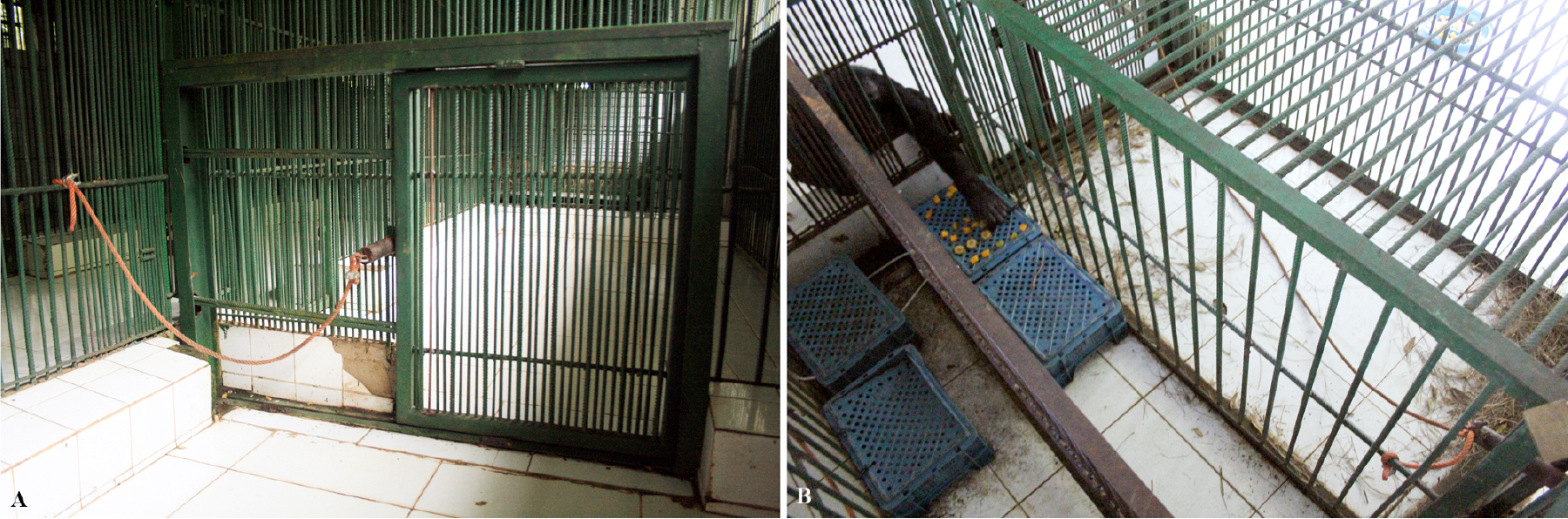

Supplement: Figure S1 — Photos of the setups of the four experiments: a) The one-way key system in experiment 1 and 2, viewed from the subject room. b) The general setup of experiment 3 and 4. This particular photo shows the subject, the baited tunnel, the divider, the one-way key in the locked position and the food as it was placed in experiment 4. (JPG) [file pone.0051922.s002.jpg]

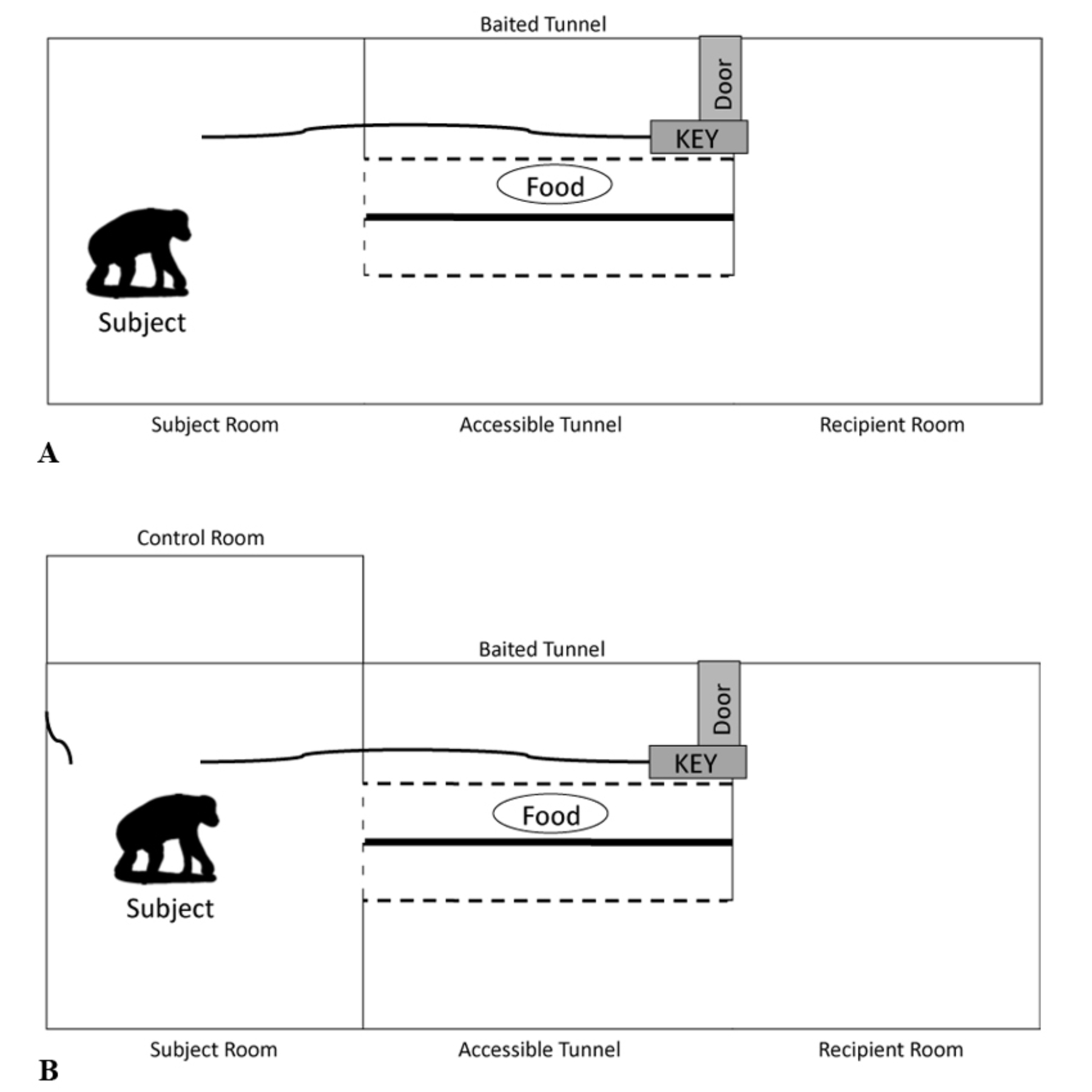

Supplement: Figure S2 — Setups of self-regard pre-test and no-food introduction of experiment 3–4: a) The self-regard pre-test of experiment 3 and 4. b) The no-food introduction of experiment 3 and 4, during which no other bonobos were present in adjacent rooms. In both phases, the subjects had to meet the corresponding criteria in five consecutive trials to proceed to the next test phase. (JPG) [file pone.0051922.s003.jpg]
