# Supplementary material for: Bonobos Share with Strangers
Source: PLoS One. 2013 Jan 2;8(1):e51922. doi: 10.1371/journal.pone.0051922 (PMC3534679; doi:10.1371/journal.pone.0051922)
Supplement: Table S1 — Subject information of experiment 1 and 2. (PDF) [file pone.0051922.s004.pdf]

Name	Sex	Age estimate[1]	Group #	Past study[2]	Experiments	Experiment 1		Experiment 2	
						S-G[3]	# of times to release each recipient[4]	Condition-Recipient[3]	# of times to unlock each room[5]
Luozi	M	7	1	No	1-2	*Waka*-Muanda	2-0	G-Muanda	3-2
Mabali	M	7	1	No	1-2	*Waka*-Lisala	5-0	S-*Kinshasa*	1-1
Kasongo	M	8	1	Yes	1-2	*Waka*-Kalina	5-0	G-Kalina	1-2
Dilolo	M	9	1	No	1-2	*Sake*-Muanda	5-0	S-*Kinshasa*	3-0
Matadi	M	10	1	No	1	*Sake*-Kalina	0-0	N/A	N/A
Kikwit	M	12	1	Yes	1	*Kinshasa*-Lisala	0-0	N/A	N/A
Muanda	F	6	1	No	1-2	Kinshasa-Kalina	4-0	G-Kalina	0-3
Lisala	F	9	1	Yes	1-2	*Kinshasa*-Muanda	4-0	S-*Masisi*	1-0
Kalina[6]	F	12	1	Yes	1-2	*Sake*-Lisala	4-1	G-Bandundu[6]	1-1
Masisi	F	4	0	No	1-2	Muanda-Waka	3-2	G-Kinshasa	4-1
Waka	F	4	0	No	1-2	*Kalina*-Kinshasa	1-4	G-Sake	4-1
Sake	F	5	0	Yes	1-2	Muanda-Masisi	0-5	S-*Bandundu*	4-0
Katako[6]	F	6	0	No	1-2	Muanda-Masisi	4-1	S-Muanda	5-0
Kinshasa	F	6	0	No	1-2	Kalina-Sake	0-1	S-*Bandundu*	3-1

1 Age estimates are based on [38] and the medical records of Lola ya Bonobo

2 Past study refers to [10]

3 S, G stands for stranger and groupmate. Names in italic refer to complete strangers.

4 The first and the second number refers to # of trials where the stranger and the groupmate was firstly released, respectively

5 The first and the second number refers to # of trials where the recipient and empty room was firstly unlocked, respectively

6 Kalina was tested with her dependent infant and Katako was tested with another 3-year-old orphan.
