# Supplementary material for: Bonobos Share with Strangers
Source: PLoS One. 2013 Jan 2;8(1):e51922. doi: 10.1371/journal.pone.0051922 (PMC3534679; doi:10.1371/journal.pone.0051922)
Supplement: Table S2 — Subject information of experiment 3 and 4. (PDF) [file pone.0051922.s005.pdf]

Name	Sex	Age estimate[1]	Group #[2]	Experi-ments	Experiment 3		Experiment 4	
					Stranger[3,4]	Groupmate[4]	Condition-Recipient[3]	# of times to pull the rope[4]
Mabali	M	8	1	3-4	*Sake* (4-1)	Masisi (0-1)	G-Kasongo	0-0
Kasongo	M	9	1	3	*Lukuru* (3-1)	Masisi (6-4)	N/A	N/A
Dilolo	M	10	1	3-4	*Chibombo* (2-3)	Kasongo (1-0)	S-Sake	0-0
Kikwit	M	13	1	3-4	*Chibombo* (1-0)	Mabali (5-4)	G-Waka	0-0
Chibombo	M	4	0	3	*Sake* (5-0)	Lukuru (4-1)	N/A	N/A
Lisala	F	10	1	3-4	*Chibombo* (1-0)	Mabali (3-1)	G-Waka	0-0
Masisi	F	5	1	3	Lukuru (0-0)	Waka (0-0)	N/A	N/A
Waka	F	5	1	3-4	Chibombo (4-1)	Kasongo (6-1)	S-Sake	0-0
Sake	F	6	2	3-4	*Kasongo* (1-1)	N/A	S-*Matadi*	0-1
Katako	F	7	1	3-4	Sake (3-0)	Masisi (4-1)	S-Chibombo	0-0

1 Age estimates are based on [38] and the medical records of Lola ya Bonobo.

2 In the year between experiment 2 and 3, Katako, Masisi, Sake and Waka have been transferred to a new group.

3 S, G stands for stranger and groupmate. Names in italic refer to complete strangers.

4 The 1st and 2nd number refers to # of trials where the rope was pulled in the experimental and control condition, respectively
