# Supplementary material for: Bonobos Share with Strangers
Source: PLoS One. 2013 Jan 2;8(1):e51922. doi: 10.1371/journal.pone.0051922 (PMC3534679; doi:10.1371/journal.pone.0051922)
Supplement: Table S3 — Reproductive history of female bonobos from the sanctuary. (PDF) [file pone.0051922.s006.pdf]

Name	Estimated Birthday[1]	Date of 1st observed menstrual cycle	Age at 1st menstrual cycle	Date of 1st birth	Age at 1st birth
Bandundu	1/1/97	5/12/06	9	5/22/08	11
Isiro	1/1/98	7/19/06	8	12/7/10	12
Kalina[2]	1/1/98	2/1/06	8	4/25/07	9
Kinshasa[2]	1/1/04	2/27/12	8	N/A	N/A
Kisantu	1/1/99	1/19/06	7	2/26/07	8
Likasi	1/1/01	N/A	N/A	6/17/09	8
Lisala[2]	1/1/01	1/17/10	9	12/4/11	10
Lukaya	1/1/00	1/28/08	8	9/19/08	8
Maya	1/1/93	3/25/03	10	5/22/04	11
Noiki	1/1/99	8/13/08	9	11/21/09	10
Opala	1/1/95	1/7/04	9	10/19/04	9
Salonga	1/1/98	N/A	N/A	1/26/06	8
Sankuru	1/1/02	2/20/10	8	N/A	N/A
Semendwa	1/1/97	3/2/04	7	9/6/05	8

1 Based on the medical records of the sanctuary and [38], all were estimated to January 1st

2 Subjects of the current study
